# Evaluation of the* In Vitro* and* In Vivo* Antioxidant Potentials of* Sudarshana* Powder

**DOI:** 10.1155/2018/6743862

**Published:** 2018-04-10

**Authors:** Weerakoon Achchige Selvi Saroja Weerakoon, Pathirage Kamal Perera, Dulani Gunasekera, Thusharie Sugandhika Suresh

**Affiliations:** ^1^Department of Ayurveda Paediatrics, Institute of Indigenous Medicine, University of Colombo, Colombo, Sri Lanka; ^2^Department of Ayurveda Pharmacology and Pharmaceutics, Institute of Indigenous Medicine, University of Colombo, Colombo, Sri Lanka; ^3^Department of Paediatrics, Faculty of Medical Sciences, University of Sri Jayewardenepura, Nugegoda, Sri Lanka; ^4^Department of Biochemistry, Faculty of Medical Sciences, University of Sri Jayewardenepura, Nugegoda, Sri Lanka

## Abstract

*Sudarshana* powder (SP) is one of the most effective Ayurveda powder preparations for paediatric febrile conditions. The objective of the present study was to evaluate the* in vitro *and* in vivo *antioxidant potentials of SP. The* in vitro *antioxidant effects were evaluated using ABTS radical cation decolourization assay where the TROLOX equivalent antioxidant capacity (TEAC) was determined. The* in vivo* antioxidant activity of SP was determined in Wistar rats using the Lipid Peroxidation (LPO) assay in serum. The* in vitro* assay was referred to as the TROLOX equivalent antioxidant capacity (TEAC) assay. For the* in vivo* assay, animals were dosed for 21 consecutive days and blood was drawn to evaluate the MDA level. The* in vitro *antioxidant activity of 0.5 *μ*g of SP was equivalent to 14.45 *μ*g of standard TROLOX. The percentage inhibition against the radical formation was 50.93 ± 0.53%. The SP showed a statistically significant (*p* < 0.01) decrease in the serum level of thiobarbituric acid-reactive substance in the test rats when compared with the control group. These findings suggest that the SP possesses potent antioxidant activity which may be responsible for some of its reported bioactivities.

## 1. Introduction

*Sudarshana powder* (SP) is the most effective antipyretic Ayurvedic preparation, widely used in Sri Lanka as well as in India from the inception of Ayurveda treatment. SP is mentioned in the Sri Lankan Ayurvedic Pharmacopeia [1] complied under Sec. 41(2) (c) of the Ayurveda Act no. 31 of 1961. The powder is prepared using different parts of plant materials and therefore is a 100% herbal product.

SP contains 53 [1] bitter ingredients and has the capability to treat fever associated symptoms such as dyspepsia, anorexia, fatigue, and nausea. It does not cause constipation and produces a mild laxative effect. It promotes the flow of bile and in gastrointestinal disorders it is used as a digestive.

Initially the main ingredient of the SP in Sri Lanka was* Swertia chirata *(Roxb. ex Fleming) which was later replaced by* Andrographis paniculata* (Burm. f.) Wall. ex Nees. Presently the SP contains* Andrographis paniculata* (Burm. f.) Wall. ex Nees. (50%) along with 52 other ingredients (50%). The other constituents too have different therapeutic uses [2].

Although literature suggests that SP is the most effective and popular Ayurvedic medicine, no scientific evidence is available for its antioxidant potential. Antipyretic effect [3–6], anti- inflammatory effect [7], analgesic effect [8–10], antihistamine effect [11], and long term administration effect [12–15] of the SP were established in Wistar rats in our recent experimental studies. Thus, in the present study, in vitro and in vivo antioxidant potentials of SP were evaluated.

## 2. Materials and Methods

### 2.1. Preparation of* Sudarshana* Powder

SP (Table 1) was prepared according to Ayurveda Pharmacopeia at the Pharmacy of the Institute of Indigenous Medicine, University of Colombo [2].

All the ingredients were purchased from Ayurveda Drug Corporation, Sri Lanka, and authentication of ingredients was done at the Institute of Indigenous Medicine, University of Colombo, Sri Lanka (Specimen No. 102), and SP was prepared according to Ayurveda Pharmacopeia [1] at the Pharmacy of the Institute of Indigenous Medicine.

All the 53 Ingredients were thoroughly cleaned to remove any contaminated materials using tap water. Washed ingredients were air-dried. Some herbal ingredients (i.e.,* Plumbago indica* Linn.) were purified using purification methods, mentioned in Ayurveda authentic texts, prior to being powdered. All ingredients were powdered at the mesh size of 80 under the Ayurveda concepts.* Andrographis paniculata* (Burm. f.) Wall. ex Nees. (2600 g) was mixed with 50 g each of the rest of the ingredients (50 × 52) to obtain the* Sudarshana* powder.

### 2.2. Animals

Healthy adult male Wistar rats (200–250 g) were used in the* in vivo* study. The animals were kept in plastic cages (two per cage) under standardized animal house conditions (temperature, 28–31°C; photoperiod, approximately 12 h natural light “per day”; relative humidity, 50–55%) at the Faculty of Medical Sciences, University of Sri Jayewardenepura, with continuous access to pelleted feed and tap water.

All experiments in rats were carried out in accordance with the guidelines for care and use of laboratory animals and the project proposal was approved (No. 591/11) by the Ethics Review Committee of the Faculty of Medical Sciences of the University of Sri Jayewardenepura, Sri Lanka (http://medical.sjp.ac.lk/index.php/ethics-review-committee-introduction).

### 2.3. *In Vitro* Antioxidant Activity

The* in vitro *antioxidant free radical scavenging activity of SP was determined by using ABTS radical cation decolourization assay [16].

In this assay, 2,2′-azinobis-(3-ethylbenzothiazoline-6-sulfonic acid) or ABTS (C_18_H_18_N_4_O_6_S_4_) is converted to its radical cation. This radical cation is dark green in colour and absorbs light at 734 nm. [16]. The reaction was monitored spectrophotometrically. This assay is often referred to as the TROLOX equivalent antioxidant capacity (TEAC) assay.

#### 2.3.1. Preparation of Extracts

The SP (5 g) was extracted with 100 ml ultrapure water, at 80°C for 20 min in a water bath shaker. After cooling, the extract was centrifuged at 5000 rpm for 10 min. The solution was filtered using No. 1 Whatman filter paper and used for ABTS analysis. The sample was diluted 1 : 10 (100 *μ*l sample + 900 *μ*l H_2_O) [17].

#### 2.3.2. Formation of ABTS Radical Solution

The ABTS solution (7 mmol) stock solution, 2.6 ml, was mixed with 11.5 ml of potassium persulfate (K_2_S_2_O_8_) solution and kept in a dark place at room temperature (23°C) for 16 hours for free radical formation. The generated ABTS radical cations (ABTS^+^) solution was dark green in colour. The stock solution was diluted with ultrapure water until the absorbance reached 0.700 (±0.02) at 734 nm. The prepared sample (10 *μ*l) was added to ABTS^+^ solution (2990 *μ*l) with phosphate saline buffer until total volume was reached (3 ml). The absorbance reading was taken 6 min after initial mixing. All determinations were performed in triplicate.

#### 2.3.3. Standard Curve for ABTS Radical (ABTS^+^) Activity on TROLOX

TROLOX (C_14_H_18_O_4_) or (6-hydroxy-2,5,7,8-tetramethylchroman-2-carboxylic acid) is equivalent antioxidant capacity (TEAC) which measures the antioxidant capacity of a given substance, as compared to the standard, TROLOX.

The stock solution of TROLOX was prepared by dissolving 0.0161 g in 50 ml deionized water. From the prepared stock solution, 5 *μ*l (1.56 *μ*g TROLOX), 10 *μ*l (3.12 *μ*g TROLOX), 15 *μ*l (4.68 *μ*g TROLOX), 20 *μ*l (6.24 *μ*g TROLOX), and 25 *μ*l (7.8 *μ*g TROLOX) were taken and reacted with ABTS^+^ solution with initial absorbance of 0.700 at 734 nm against a phosphate saline buffer blank. Absorbance of reaction mixture was taken till the absorbance came to a plateau. The reduction of absorbance was calculated from the initial and final absorbance. The reduction of absorbance relevant to each TROLOX concentration was performed six times. Standard curve was drawn for mean reduction of absorbance versus quantity of TROLOX in *μ*g. The antioxidant activity was expressed as TROLOX equivalent antioxidant capacity (TEAC).

### 2.4. *In Vivo* Antioxidant Activity

*In vivo* antioxidant activity of SP was analysed using the method of determination of the Lipid Peroxidation (LPO) in serum. The level of thiobarbituric acid-reactive substance (TBARS) and malondialdehyde (MDA) production was measured in serum by the modified method by Draper and Hadly, 1990 [18].

Wistar rats were randomly divided into two groups of six animals each. On Day 0, blood samples were collected to assess the baseline serum malondialdehyde (MDA) level. The control group received distilled water and test group received hot water extraction of SP (0.5 g/kg). The animals were dosed for 21 consecutive days and were observed daily for signs of toxicity and death throughout the period of study. Body weights were recorded. Twenty-four hours after the last treatment, blood was obtained through direct cardiac puncture to evaluate the MDA level.

The serum (200 *μ*L) was deproteinized by adding 1 ml of 14% trichloro acetic acid and 1 ml of 0.6% thiobarbituric acid. The mixture was heated in a water bath for 30 min at 95°C to complete the reaction and then cooled on ice for 5 min. Following centrifugation at 2000 rpm for 10 min, the absorbance of the coloured product (TBARS) was measured at 535 nm with a UV spectrophotometer.

The TBARS concentration was calculated using the following formula:

(1) $$\begin{array}{c} A=\Sigma CL, \end{array}$$

where

 
*A* is absorbance,
 
Σ is molar coefficient (1.56 × 10^5^ L/mol/cm),
 
*C* is concentration of sample,
 
*L* is path length (1 cm).

#### 2.4.1. Statistical Analysis

The results were analysed using “Student's *t*-test.” Values of *p* < 0.05 were considered statistically significant.

## 3. Results

### 3.1. *In Vitro* Antioxidant Activity

The standard curve equations *y* = 0.040*x* + 0.060 and *R*^2^ = 0.9962 were obtained from the standard curve for TROLOX. The antioxidant activity of SP 0.5 *μ*g was equivalent to 14.45 *μ*g of standard TROLOX. The percentage inhibition against the radical formation was 50.93 ± 0.53%.

### 3.2. *In Vivo* Antioxidant Activity

Each value represents the mean ± SEM of MDA concentration. Values carrying different superscripts are significantly different (^*∗*^*p* < 0.05, ^*∗∗*^*p* < 0.01, and ^*∗∗∗*^*p* < 0.001).

The findings of the study in the rats tested: the serum MDA levels of the control and test group (SP), respectively, were 3.9 ± 0.21 *μ*mol/L and 2.07 ± 0.08 *μ*mol/L (*p* < 0.01) and significant reduction (*p* < 0.001) of serum MDA level was observed on Day 21 when compared with Day 0 level of the test group; moreover there were no differences observed in the control group's MDA concentration on Day 0 and Day 21 (Table 2).

## 4. Discussion

An antioxidant is a substance that is able to protect a substrate susceptible to oxidation, being itself present at fairly low concentrations in relation to the substrate. The SP possesses significant therapeutic effects but scientific evidence for these benefits is scarce. Therefore the* in vitro* and* in vivo* antioxidant activity of SP were evaluated in this study using ABTS and TBARS assays, respectively.

The ABTS radical method is one of the most frequently used assays for the determination of the concentration of free radicals. The method is applicable to the study of both water-soluble and lipid-soluble antioxidants, pure compounds, and food extracts. The results of the ABTS assay done with SP explained without doubt the potent ability to neutralize the radical spontaneously. The antioxidant activity of 0.5 *μ*g of SP was equivalent to 14.45 *μ*g of standard TROLOX. The percentage inhibition against the radical formation was 50.93 ± 0.53% and confirms the strong antioxidant power of the SP.

The thiobarbituric acid-reactive substances (TBARS) assay is a widely used method to quantify the concentration of MDA in serum, plasma, or tissue homogenates. At low pH and elevated temperature, MDA readily participates in a nucleophilic addition reaction with 2-thiobarbituric acid (TBA), generating a pink and fluorescent 1 : 2 MDA : TBA adduct. It is a simple, reliable, and a reproducible fluorometric method for measuring TBARS in samples [19]. In this study, the SP showed the significant scavenging activity against MDA formation in rats providing evidence for the potent antioxidant activity of the SP.

## 5. Conclusion

The present investigation suggests that poly herbal preparation of* Sudarshana* powder possesses good antioxidant potential and it can be a useful therapeutic agent for the diseases associated with oxidative stress.

## Figures and Tables

**Table 1:** Ingredients of *Sudarshana* powder.

Botanical name	General user name in Sri Lanka	Sanskrit name	Parts used
(1) *Andrographis paniculata* (burm. f.) Wall. ex Nees.	Heen bincohomba	Chirayetah	Whole plant
(2) *Piper longum* Linn.	Thippili mul	Chapala	Root
(3) *Zingiber officinale* Rosc.	Inguru	Ardraka	Rhizome
(4) *Piper nigrum* Linn.	Gammiris	Katuka	Fruit
(5) *Piper longum* Linn.	Thippili	Chapala	Fruit
(6) *Santalum album *Linn.	Suduhandun	Chandana	Stem
(7) *Glycyrrhiza glabra* Linn.	Valmi	Yashtimadu	Stem
(8) *Picrorhiza kurroa *Royle ex Benth.	Katukarosana	Katurohini	Herb
(9) *Cedrus deodara* (Roxb.) Loud.	Devađara	Devodara	Timber
(10) *Hedychium spicatum* (Ham. ex Smith)	Hingurupiyali	Shati	Rhizome
(11) *Embelia ribes* Burm F.	Valanghasal	Krimigna	Fruit
(12) *Trachyspermum roxburghianum* (DC.) Craib.	Asamodagam	Ugragandha	Whole herb
(13) *Holarrhena antidysenterica *Wall.	Kelinda	Kutaja	Bark and wood
(14) *Aconitum heterophyllum* Wall.	Athividayan	Athivida	Root
(15) *Terminalia chebula* Retz.	Aralu	Abaya	Dry fruit
(16) *Terminalia bellirica* (Gaertner) Roxb.	Bulu	Vibhitaka	Dry fruit
(17) *Phyllanthus emblica* Linn.	Nelli	Amalakee	Dry fruit
(18) *Syzygium aromaticum* (L.) Merr. & Perry	Karabunati	Lavanga	Flower
(19) *Myristica fragrans* Linn.	Vasāvāsi	Jathipala	Fruit cover
(20) *Cyperus rotundus *Linn.	Kalānduru ala	Granthi	Rhizome
(21) *Solanum melongena* Linn.	Elabatumul	Vruhathi	Plant
(22) *Alysicarpus vaginalis* (L.) DC.	Asvanna	-	Plant
(23) *Aerva lanata* (L.) Juss. ex Schult.	Polpala	-	Whole plant
(24) *Solanum xanthocarpum *Schrad. & Wendl.	Katuvelbatu	Brihati	Plant
(25) *Saussurea lappa* Linn.	Suvandakottan	Kushta	Root
(26) *Azadirachta indica* A. Juss.	Kohambapothu	Nimba	Bark
(27) *Tinospora cordifolia* (Willd.) Miers ex Hook. f. & Thoms.	Rasakinda	Amurtha	Stem
(28) *Curcuma domestica* Valet.	Kaha	Haridra	Rhizome
(29) *Cinnamomum iners* Reinw.	Kollan kola	Thejapatra	Leaves
(30) *Trichosanthes cucumerina* Linn.	Dummalla	Patola	Leaves
(31) *Tragia involucrata* Linn.	Val kahabilia	Duralaba	Root
(32) *Marsdenia tenacissima* (Roxb.) Moon.	Murva	Tejowapi	Stem
(33) *Oldenlandia biflora* Linn.	Pathpadagam	Parpataka	Whole plant
(34) *Bacopa monniera* (L.) Wettst.	Lunuvila	Brahmi	Whole plant
(35) *Plectranthus zeylanicus* Benth.	Irivriya	Valakan	Whole plant
(36) *Holarrhena antidysenterica* (Linn.) Wall.	Kelinda hal	Kutaja	Seeds
(37) *Moringa oleifera* Lam.	Murunga eta	Shigruka	Whole plant
(38) *Cinnamomum zeylanicum* Blume.	Kurundupothu	Bahugandha	Bark
(39) *Abies webbiana* Lindl.	Thalispatra	Thalispatra	Leaves
(40) *Nelumbium speciosum* Willd.	Padma kashta	Padmakashta	Rhizome
(41) *Vetiveria zizanioides* (L.) Nash.	Savandarā	Ushira	Root
(42) *Sida cordifolia* Linn.	Bavilamul	Bala	Root
(43) Aluminium sulphate	Seenakkaran	Surashtaja	
(44) *Valeriana wallichii* DC.	warala	Tagar	Root
(45) *Plumbago indica* Linn.	Rathnitol	Chitraka	Root
(46) *Coscinium fenestratum *(Gaertn.) Colebr.	Wenivalgata	Pitadaru	Wood, stem
(47) *Premna herbacea* Roxb.	Sirithekku	Bhangi	Root
(48) *Asparagus racemosus *(Willd.) Oberm.	Kavelu (Hatavariya)	Shathavari	Rhizome
(49) *Bambusa vulgaris *Schrad ex Wendl.	Unakapuru	Vamash	Extraction
(50) *Withania somnifera* Linn.	Rishibaka (Amukkara)	Ashvagandha	Rhizome
(51) *Piper chawya *Buch.-Ham.	Siviya	Siviya	Root
(52) *Nelumbium speciosum* Willd.	Nelum	Pundarika	Lotus stamens
(53) *Ipomoea digitata* Linn.	KiribaduAla	Jeevaka	Rhizome

**Table 2:** Effect of drugs on lipid peroxidation in Wistar rats.

Rat group	MDA con. (*µ*mol/L)	
	Day 0	Day 21
Control	3.8 ± 0.06	3.9 ± 0.21
Test SP	3.7 ± 0.05	2.07 ± 0.08

Values are expressed as mean ± SEM; *n* = 6 per group.
